# Chronic Pancreatitis and Pregnancy: Genetics Matter

**DOI:** 10.14309/ctg.0000000000000698

**Published:** 2024-04-25

**Authors:** Yasmin G. Hernandez-Barco, Julia McNabb-Baltar

**Affiliations:** 1 Division of Gastroenterology, Massachusetts General Hospital, Boston, MA;; 2 Division of Gastroenterology, Brigham and Women's Hospital, Boston, MA.

In this issue of *Clinical Translational Gastroenterology*, the study by Wu et al (1) represents a significant contribution to our understanding of chronic pancreatitis (CP) during pregnancy. Specifically, they examined a large prospective cohort study of pregnant patients with CP to explore the impact of genetic mutations associated with CP on pregnancy outcomes. They included 160 female patients with CP and history of pregnancy. Of these, 59.4% of patients had pathogenic mutations in CP-susceptibility genes (*SPINK1*, *PRSS1*, *CTRC*, and *CFTR*).

Prior studies have shown that patients with CP have an increased risk of gestational diabetes (adjusted odds ratio [AOR] 1.63, 95% CI 1.19–2.23), gestational hypertension (AOR 2.48, 95% CI 1.87–3.29), and preterm labor (AOR 3.10, 95% CI 2.40–4.00) and low risk for gestational age (AOR 2.40, 95% CI 1.35–3.08) (2). Wu et al extend this knowledge, uncovering that CP-susceptible genetic mutations exacerbate the risk of adverse pregnancy outcomes. The authors identified that of 364 pregnancies and 227 deliveries, CP-associated adverse pregnancy outcomes occurred in 23.8% of patients. Of these, patients with gene mutations in the CP-susceptibility genes had higher incidence of CP-associated adverse pregnancy outcomes compared with those without mutations (30.5% vs 13.8%, *P* = 0.015). The incidence of overall adverse maternal outcomes was higher in patients with gene mutations (20.0% vs 9.2%), but this was not statistically significant (*P* = 0.065). However, the number of CP-associated preterm deliveries was significantly higher in patients with genetic mutations (12.6% vs 3.1%, *P* = 0.036). In addition, the rate of adverse fetal outcomes was significantly higher when born from mothers with genetic mutations (22.1% vs 9.2%, *P* = 0.033), as was the rate of CP-associated abortions (17.9% vs 4.6%, *P* = 0.013). These findings suggest the importance of screening and testing patients for genetic mutations if there is no clear etiology for their pancreatitis as the presence of a known genetic mutation can increase the risk of adverse outcomes.

The study's findings challenge previous assumptions and contradict some earlier research suggesting no significant difference in pregnancy outcomes between women with CP and those without (3,4). These studies, however, assessed very different cohorts; the study by Rana et al compared all patients with CP with those without CP and the study by Wu et al compared CP patients with and without genetic mutations. In addition, it is important to note that both studies, which did not find adverse health outcomes, included patients from India suggesting that geographic influences may play a role in CP and pregnancy outcomes.

It is noteworthy that the most common genetic mutation was *SPINK1* (57.9%), and patients with the c.194 + 2T>C variant had a significantly higher risk of adverse pregnancy outcomes when compared with those without that specific mutation. SPINK1 is required for normal embryological development, and mutations in this gene increase the risk of congenital malformations (5). *SPINK1* is by far the most common CP-susceptibility gene mutation in the Chinese population, and these findings may not be generalizable to other areas in the world. It will be important for future studies to examine if SPINK1 mutations in other regions show similar adverse pregnancy outcomes and examine the effect of other genetic mutations on pregnancy outcomes in the Americas, Europe, Africa, and other parts of Asia.

The authors also found that any acute pancreatitis attack during pregnancy greatly increased the risk of adverse pregnancy outcomes (Odds Ratio (OR) 5.63, 95% Confidence Interval (CI) 1.68–18.87). The authors have identified 2 unique risk factors not previously reported. The presence of the *SPINK1* mutation and an episode of acute pancreatitis during pregnancy both increased the risk of adverse pregnancy outcomes. This suggests that patients with known genetic mutations should be closely monitored during pregnancy and that all measures to avoid recurrent episodes of pancreatitis during pregnancy should be taken.

Interestingly, all patients with exocrine pancreatic insufficiency were treated with pancreatic enzyme replacement therapy, and the presence of exocrine pancreatic insufficiency did not affect pregnancy outcomes. Similarly, diabetes related to CP did not correlate with an increased risk of adverse pregnancy events in this study. Nonetheless, these findings underscore the importance of diligent monitoring and management of both diabetes and exocrine pancreatic insufficiency in pregnant patients. Such proactive care is crucial to circumvent potential complications, ensuring a safer pregnancy journey.

This study represents the first to carefully delineate the experiences of pregnant patients with CP, distinguishing between those with and without known genetic mutations in CP-susceptibility genes. While previous research has documented a rise in adverse pregnancy outcomes associated with CP, the specific influence of genetic mutations on these outcomes had not been explored until now. In summary, this study is quite impactful at the intersection of pregnancy and pancreatitis research as it uncovered the importance of CP-susceptibility genes on pregnancy outcomes. It underscores the need for genetic counseling and testing among patients with CP contemplating pregnancy, given the elevated risk of complications for those carrying genetic mutations.

## References

[1] Wu D Ru N Wang Y-C Genetic factors associated with adverse pregnancy outcomes in chronic pancreatitis. Clin Transl Gastroenterol 2024. doi. 10.14309/ctg.0000000000000691 PMC11042769 38334943

[2] Niu C Zhang J Zhu K The hidden dangers of chronic pancreatitis in pregnancy: Evidence from a large-scale population study. Dig Liver Dis 2023;55(12):1712–8. 37474413 10.1016/j.dld.2023.07.001

[3] Rana A Sharma S Qamar S Chronic pancreatitis in females is not associated with adverse pregnancy outcomes: A retrospective analysis. J Clin Gastroenterol 2023;57(5):531–6. 35470319 10.1097/MCG.0000000000001711

[4] Mahapatra SJ Midha S Teja GV Clinical course of chronic pancreatitis during pregnancy and its effect on maternal and fetal outcomes. Am J Gastroenterol 2021;116(3):600–8. 33657043 10.14309/ajg.0000000000001076

[5] Kolho KL. Kazal type trypsin inhibitor in amniotic fluid in fetal developmental disorders. Prenat Diagn 1986;6(4):299–302. 3748994 10.1002/pd.1970060410

